# Clinical Applicability of Artificial Intelligence–Driven Implant Planning and Surgical Guide Design in the Maxillary Esthetic Zone: A Registry‐Based Cohort Study

**DOI:** 10.1111/clr.70144

**Published:** 2026-06-03

**Authors:** Eslam Abdelwahab Dawood, Bahaaeldeen M. Elgarba, Rocharles Cavalcante Fontenele, Reinhilde Jacobs

**Affiliations:** ^1^ OMFS‐IMPATH Research Group, Department of Imaging and Pathology Faculty of Medicine, KU Leuven Leuven Belgium; ^2^ Department of Prosthodontics, Faculty of Dentistry Tanta University Tanta Egypt; ^3^ Department of Oral and Maxillofacial Surgery University Hospitals Leuven Leuven Belgium; ^4^ Department of Stomatology, Public Health and Forensic Dentistry, Division of Oral Radiology, School of Dentistry of Ribeirão Preto University of São Paulo (USP) Ribeirão Preto Brazil; ^5^ Department of Dental Medicine Karolinska Institute Stockholm Sweden

**Keywords:** artificial intelligence, cone beam computed tomography, dental implants, digital dentistry, esthetic zone, implant planning

## Abstract

**Objectives:**

This study evaluated the accuracy, time efficiency, and workflow consistency of artificial intelligence (AI)‐assisted versus human expert (HI) implant planning in the esthetic anterior maxilla.

**Material and Methods:**

Thirty‐five single‐tooth anterior maxillary cases with paired cone beam computed tomography (CBCT) and intraoral scans (IOS) were retrospectively recruited. A hybrid AI framework (Relu Automate), integrating rule‐based constraints (≥ 2 mm labial bone, ≥ 1 mm palatal bone) with deep learning segmentation, was compared with conventional HI. The performance of each planning approach was evaluated based on the following: coronal and apical linear deviations, angular deviation, bone thickness, wax‐up alignment, surgical guide fit, planning time, and consistency. Statistical analyses were performed with a significance level of 5% (α = 0.05).

**Results:**

AI‐assisted planning demonstrated clinically acceptable accuracy with mean coronal, apical, and angular deviations of 0.96 ± 0.6 mm, 1.24 ± 0.66 mm, and 4.3° ± 2.9°, respectively, meeting clinical thresholds in 94.3% (coronal), 85.7% (apical), and 94.3% (angular) of cases. Bone thickness measurements were equivalent between groups (labial: 2.0 mm for both groups; palatal: 1.5 mm AI versus 1.3 mm HI; *p* > 0.05). AI‐assisted planning decreased the median planning time by 41.4% (*p* < 0.0001) while achieving perfect consistency compared to human experts (*p* < 0.05). ICC analysis revealed excellent agreement (≥ 0.9) across all spatial coordinates between AI and HI planning.

**Conclusions:**

AI‐assisted implant planning achieved accuracy comparable to expert planning with improved efficiency and consistency, while AI‐designed surgical guides demonstrated fit comparable to human‐designed guides in the maxillary esthetic zone.

## 1 Introduction

Planning dental implants in the anterior maxilla poses unique challenges due to its esthetic sensitivity and anatomical complexity. Even minor positional deviations can compromise facial harmony, necessitating precise anatomical‐prosthetic integration for predictable outcomes (Flügge et al. 2022). Thin labial plates, ridge concavities, and nasopalatine canal proximity render planning highly operator‐dependent and experience‐intensive (Dong et al. 2025).

Digital workflows have transformed this process by integrating 3‐dimensional (3D) cone‐beam computed tomography (CBCT) and intraoral scans (IOS) to enable prosthetically driven, patient‐specific positioning (Preda et al. 2024; Elgarba, Fontenele, Ali, et al. 2024). These datasets allow clinicians to optimize implant dimensions, anticipate bone‐restoration relationships, and identify augmentation needs preoperatively (Dhondt et al. 2022; Elgarba, Fontenele, Tarce, and Jacobs 2024; Gönüldaş et al. 2025).

Guided implant surgery further enhances predictability and safety by transferring virtual plans into clinical practice (Vercruyssen et al. 2015). Static guides precisely direct drilling to control angulation and depth (Huang et al. 2023), yet accuracy remains vulnerable to CBCT artifacts, IOS‐CBCT misregistration, outdated libraries, suboptimal design, 3D‐printing tolerances, resin warpage, and mucosal thickness variations (Unsal et al. 2020; Andrade‐Bortoletto et al. 2025).

Artificial intelligence (AI) now offers solutions to streamline these steps. AI excels in anatomical segmentation, IOS‐CBCT registration, and landmark detection, matching expert accuracy while slashing manual effort (Morgan et al. 2023; Fontenele et al. 2023; Preda et al. 2024; Elgarba, Fontenele, Ali, et al. 2024; Lawand et al. 2025). This yields precise virtual patients for detecting critical features like thin labial plates and root proximity (Jindanil et al. 2024; Jindanil, Baldini, et al. 2025; Baldini et al. 2025). AI also aids postoperative abutment and crown design (Fonseca et al. 2026; Dawood et al. 2026a).

Despite the growing integration of AI‐driven tools in dental implant planning (Elgarba, Fontenele, Mangano, and Jacobs 2024; Elgarba, Ali, Fontenele, et al. 2025; Elgarba, Fontenele, Du, et al. 2025; Mangano et al. 2024; Alqutaibi et al. 2025; Roongruangsilp et al. 2025), clinical validation of AI‐assisted planning workflows in the anterior maxilla remains limited. This anatomical region presents challenges related to esthetics, limited bone availability, and proximity to anatomical structures, making clinician expertise essential. Given the traditionally operator‐dependent nature of treatment planning in this area, exploring how AI can help establish a standardized initial proposal that clinicians can review and refine is clinically relevant.

Accordingly, the present study aimed to evaluate the clinical feasibility, accuracy, and efficiency of AI‐assisted implant planning for single‐tooth replacement in the anterior maxilla. The methodology compared AI‐generated preliminary plans, serving as a decision‐support input, with final plans optimized by experienced clinicians (HI). The objectives were to assess key performance metrics, including positional accuracy, surgical guide design, planning time, and inter‐case consistency, to determine how AI may enhance planning standardization within a clinician‐supervised workflow.

## 2 Materials and Methods

### 2.1 Study Design

This study was conducted as a registry‐based observational study (Joda et al. 2026) comparing AI‐assisted and HI virtual implant planning and surgical guide design for single‐tooth replacement in the anterior maxilla. This study was conducted and reported in accordance with the STROBE reporting guidelines and the recommendations for AI studies in dentistry (Schwendicke et al. 2021).

### 2.2 Ethical Approval

The study was approved by the Local Ethics Committee (reference number: S66447) and adhered to the principles outlined in the International Conference on Harmonization Guidelines for Good Clinical Practice, the Declaration of Helsinki, the Oviedo Convention, and relevant legal requirements (World Medical Association 2013). All patient data were anonymized to ensure confidentiality.

### 2.3 Data Collection

The sample size for the proposed study was calculated using G*Power program (version 3.1.9; University of Düsseldorf, Germany) based on the coronal deviation data from a previous study (Elgarba, Fontenele, Du, et al. 2025). Assuming a paired study design with a standard deviation of 0.65 mm and an expected mean difference of 0.325 mm with effect size = 0.5, a two‐sided paired *t*‐test with a significance level (α) of 0.05 and statistical power of 0.8 was employed. Based on these parameters, a total of 35 implants were required to detect a statistically significant difference between planning approaches.

Thirty‐five cases of patients with missing maxillary anterior teeth were included. Inclusion criteria comprised a single edentulous site in the anterior maxilla (central incisors, lateral incisors, or canines), CBCT imaging performed 3–6 months after tooth extraction, and adequate bone dimensions with a minimum width of 6 mm and a minimum height of 12 mm. Exclusion criteria included CBCT scans with motion artifacts, poor image quality, insufficient bone volume at the edentulous site, recent extractions, or the presence of pathological conditions. No exclusions were applied based on gender, ethnicity, income level, or prior dental history.

A dataset consisting of 35 CBCT and IOS records of single anterior maxillary teeth (central incisors = 15, lateral incisors = 10, canines = 10) was retrospectively retrieved from the imaging database of UZ Leuven Hospital, Belgium, covering the period from 2016 to 2025. This cohort included 25 men and 10 women patients, with a mean age of 43 ± 17 years (males: 45 ± 17 years; females: 37 ± 16 years).

CBCT scans were acquired using a NewTom VGI Evo device (Cefla, Bologna, Italy) with fields of view ranging from 8 × 8 to 15 × 15 cm and voxel sizes of 0.25, 0.20, or 0.10 mm^3^. IOS data were obtained using a TRIOS 3 intraoral scanner (3Shape A/S, Copenhagen, Denmark). CBCT datasets were exported in Digital Imaging and Communications in Medicine (DICOM) format, whereas IOS datasets were exported in standard tessellation language (STL) format.

### 2.4 Implant Planning Methods

A minimum ridge width of 6 mm was used as an inclusion criterion to ensure that a clinically feasible implant could be placed within the predefined safety envelope. Implant diameter was selected case by case according to ridge width, with the primary planning objective of maintaining a target safety envelope of ≥ 2 mm labial bone and ≥ 1 mm palatal bone. These thresholds were planning targets, not mandatory constraints. In both AI‐ and HI‐driven planning, implant positions were aligned with the available bone and prosthetic wax‐up contours while maintaining a minimum distance of 1.5 mm from adjacent natural teeth and 3 mm from adjacent implants. In deficient sites, implants were positioned slightly palatally to prioritize preservation of the buccal bone and maintain clinical viability (Testori et al. 2018; Monje et al. 2019; Gamborena et al. 2021). Both workflows followed the same implant envelop criteria and safety protocol to ensure clinical viability.

Two independent operators performed implant planning using different methodologies: one operator conducted AI‐assisted planning, while a second operator performed conventional human expert planning. The operators worked independently and were blinded from each other's planning processes:

#### 2.4.1 Human Intelligence (HI) Implant Planning

To establish a high‐level control group, an expert prosthodontist (E.A.D.) performed manual planning using Atomica.ai (Atomica Technology Inc., Atlanta, USA), an accredited, widely adopted clinical software representing the current gold standard in digital implant dentistry. CBCT and IOS data were uploaded for each case, and virtual implant placement followed the same predefined clinical guidelines to optimize both bone and prosthetic structure utilization.

Although the platform did not provide fully automated anatomical segmentation for implant planning, it offered separate automated segmentation modules for teeth and bone. Registration between IOS and CBCT datasets was achieved using a semi‐automated point‐to‐point approach. Corresponding anatomical landmarks were manually selected in both datasets, after which the software computed spatial alignment followed by a best‐fit refinement to ensure registration accuracy.

**FIGURE 1:**
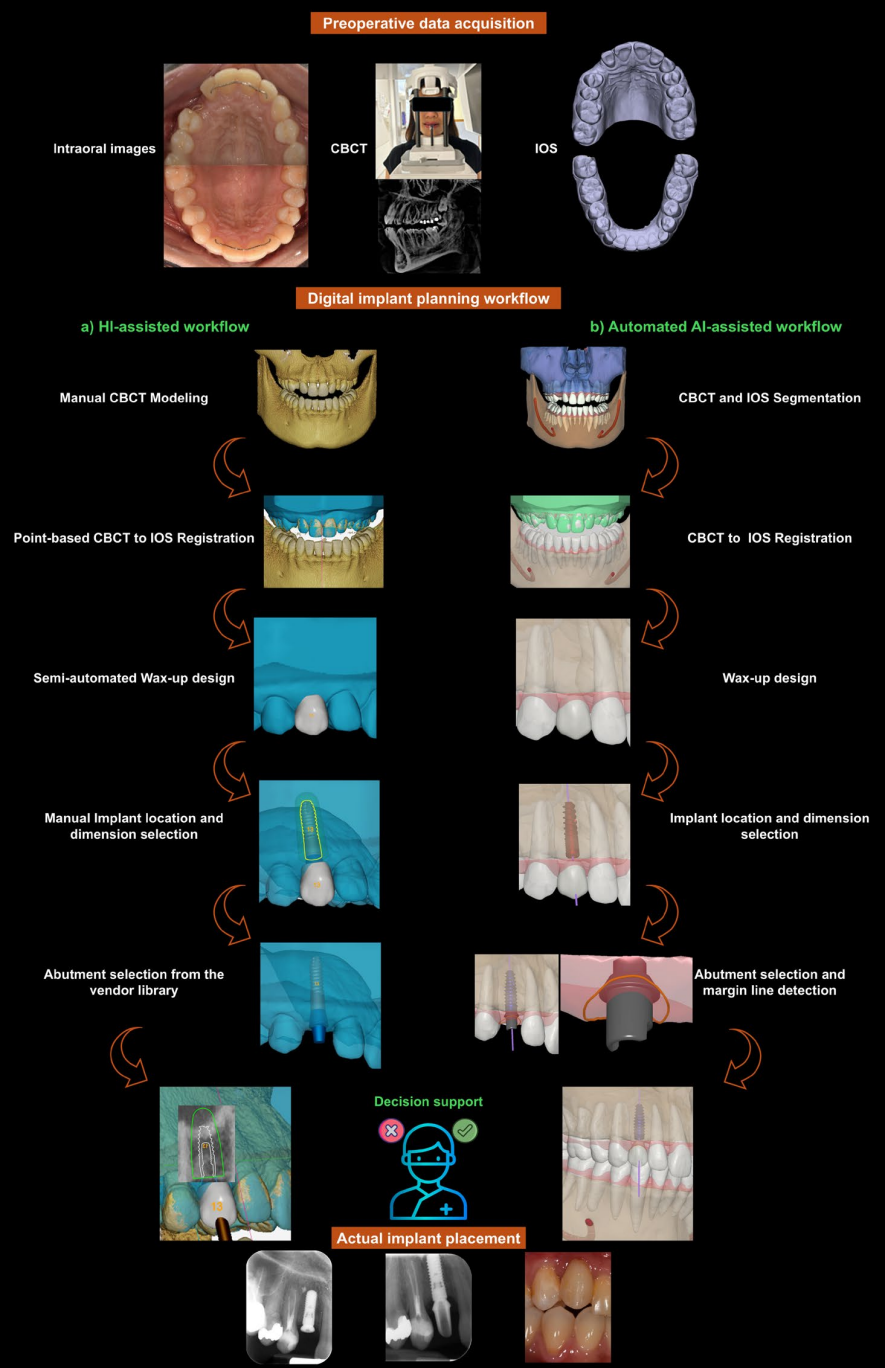
Implant planning for single‐tooth rehabilitation of the missing right upper canine (FDI tooth 13) was performed using two planning approaches: (a) Human intelligence‐based planning using Atomica.ai software program, involving a 3.8 × 12 mm implant, with the prosthetic wax‐up visualized in 3D CBCT model registered with the intraoral scan; (b) AI‐assisted automated planning in Relu Automate, which selected a 3.8 × 12 mm implant aligned with the automatically designed wax‐up on the CBCT and IOS 3D segmented model.

Subsequently, a software‐generated virtual wax‐up was created and manually refined based on the contralateral tooth morphology and overall arch morphology to achieve the desired position, size, and contour. Implant dimensions were selected according to the available bone and predefined guidelines (Testori et al. 2018; Monje et al. 2019; Gamborena et al. 2021). Virtual implant placement was performed directly on axial, coronal, and sagittal CBCT views. All implant plans were peer‐reviewed by a second expert (B.M.E.). In cases of disagreement, consensus was reached through expert discussion. Unresolved cases were referred to R.J., a radiology and implant dentistry specialist with more than 10 years of clinical experience, for final adjudication (Figure 1a).

#### 2.4.2 Artificial Intelligence (AI) Implant Planning

The same CBCT and IOS datasets were uploaded by a different operator (B.M.E.) to an AI‐driven cloud‐based platform (Relu Automate, Leuven, Belgium). This AI framework uses supervised, multiple 3D U‐networks for coarse and fine anatomical segmentation, including teeth (Lahoud et al. 2021; Ayidh Alqahtani et al. 2023), jawbones (Verhelst et al. 2021; Preda et al. 2022; Morgan et al. 2022; Fontenele et al. 2023), mandibular canals (Lahoud et al. 2022; Jindanil et al. 2023; Oliveira‐Santos et al. 2023; da Andrade‐Bortoletto et al. 2025), maxillary complex structures (Preda et al. 2022; Morgan et al. 2023), and dental implants and prosthetic crowns (Elgarba et al. 2023; Elgarba, Ali, Fontenele, et al. 2025). The platform achieved a mean dice similarity coefficient (DSC) of 0.938 (Nogueira‐Reis et al. 2024).

After automated anatomical segmentation of the CBCT and IOS scans, the two datasets were registered and refined via AI‐driven iterative closest point (ICP) alignment (Preda et al. 2024; Elgarba, Fontenele, Ali, et al. 2024). By integrating IOS‐derived crowns with CBCT‐derived roots (Baldini et al. 2025), the platform generates a high fidelity virtual patient model (Jindanil et al. 2024; Jindanil, Baldini, et al. 2025; Jindanil, Burlacu‐Vatamanu, et al. 2025). Within this digital environment, the maxillary and mandibular jaw segments are articulated into a maximum intercuspal position to optimize the subsequent prosthetic planning phase (Dawood et al. 2026b).

This platform has previously been validated for virtual implant planning in the posterior mandible in previous studies (Elgarba, Fontenele, Mangano, and Jacobs 2024; Elgarba, Fontenele, Dawood, et al. 2025; Elgarba, Fontenele, Du, et al. 2025). Implant placement was guided by machine learning restriction values informed by prior knowledge of adjacent AI‐segmented 3D anatomical structures (i.e., neighboring teeth, mandibular canal, and buccal and lingual cortical plates). The AI was trained to determine the optimal implant location and dimension by considering anatomical constraints, including maintaining a minimum distance of 2 mm from the mandibular canal, 1.5 mm from adjacent teeth, and 3 mm from adjacent implants, and labial bone thickness of at least 2 mm. Additionally, the AI aimed to achieve a compromise in the implant long axis by balancing between the automatically generated wax‐up long axis and the long axis of the adjacent teeth (Figure 1b).

For both the HI and AI planning approaches, a standardized implant from the BioHorizons digital library (BioHorizons Implant Systems Inc., Birmingham, USA) was utilized. Both workflows utilized the same clinical datasets and anatomical constraints; yet, they were intentionally designed to be distinct, thereby reflecting the human‐driven versus AI‐assisted digital planning workflow.

### 2.5 Surgical Guide Design

In the HI workflow, implant planning was followed by manual selection of the appropriate sleeve type and size according to the BioHorizons Pro Guided Surgery Kit. Surgical guides were designed using the surgical guide module in Atomica.ai software program, with standardized support extending to at least three anterior and two posterior teeth adjacent to the edentulous site. Inspection windows were incorporated when necessary to verify accurate seating intraoperatively.

The AI workflow involved a fully automated design process. The platform autonomously selected the appropriate sleeve type based on the BioHorizons Pro Guided system specified during planning and generated the surgical guide without human input.

Printing parameters were standardized as follows: tooth offset of 0.15 mm, guide thickness of 2.3 mm, and sleeve printer offset of 0.2 mm. The software program subsequently generated STL files for guide fabrication.

From each planning approach, implant positions (represented as cylindrical generic implants), prosthetic wax‐ups, and surgical guides were exported in STL format for subsequent comparative analysis.

### 2.6 Performance Assessment of AI‐Driven Implant Planning

#### 2.6.1 Implant Linear and Angular Deviation

**FIGURE 2:**
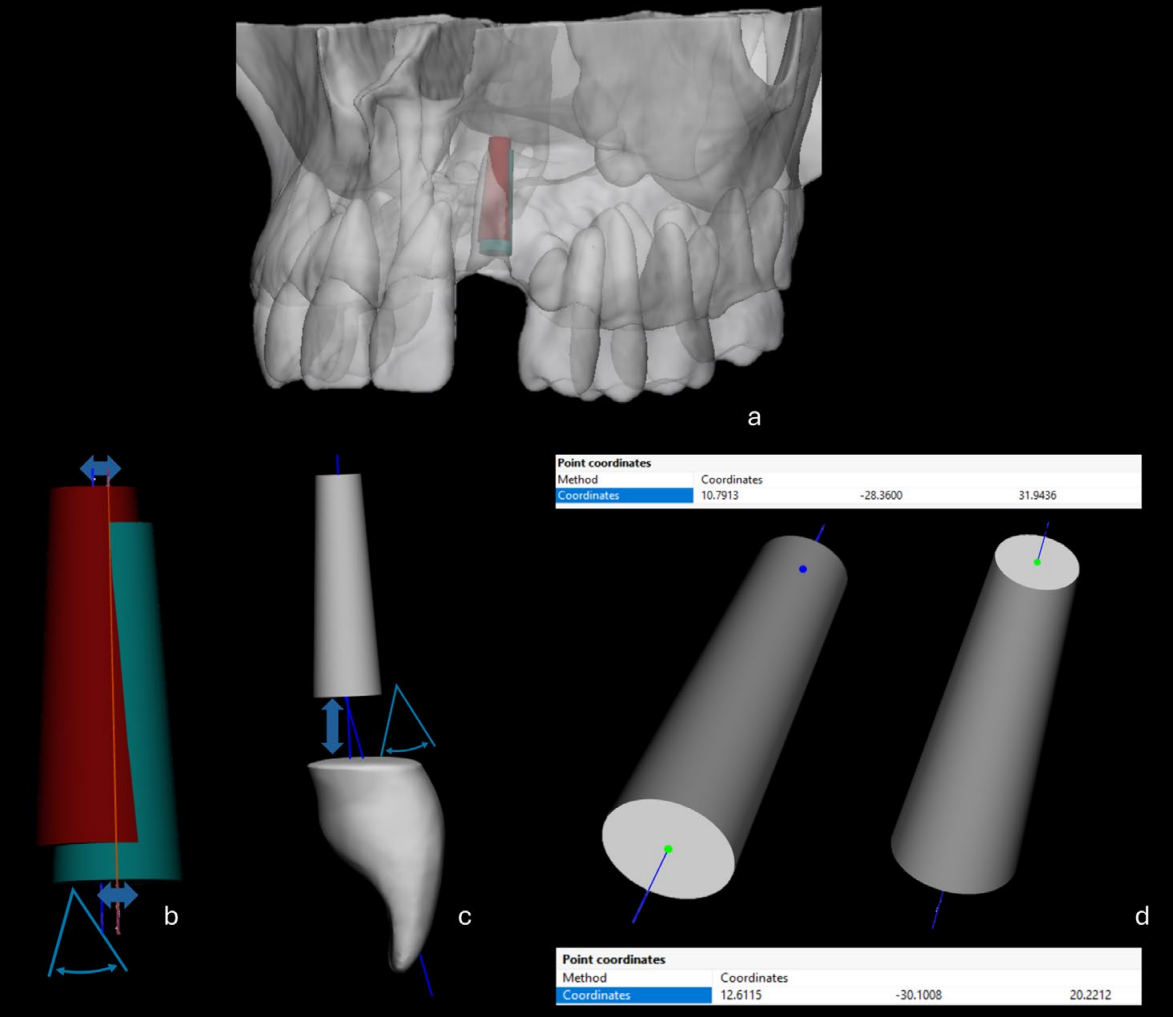
Performance assessment of AI‐driven implant planning: (a) Overlap between AI (in green) and HI (in red) planned implant within the virtual patient at 3‐Matic software program; (b) Linear and angular deviations measurements between the planned implants; (c) Wax‐up to implant angle and distance measurements; (d) Determining the coordinates of the central points of the coronal and apical implant axis.

Implants planned using both HI and AI approaches were imported into Materialise 3‐matic software program (Materialise, Leuven, Belgium). This software program automatically determined the long axis of each implant and identified intersection points at the coronal and apical surfaces. Linear deviations at the coronal and apical levels were calculated as the distances between corresponding points of HI‐ and AI‐planned implants. Angular deviation was defined as the angle between the long axes of the two implants, enabling quantitative comparison between planning methods (Figure 2b).

#### 2.6.2 Implant Planning Agreement Between AI and HI

3D implant positions were recorded using the Design Analytical Point tool in Materialise 3‐Matic (Materialise, Leuven, Belgium). The centers of the coronal platform and apical tip were identified along the implant axis to define spatial positions. Cartesian coordinates (x, y, z) for coronal and apical points were exported for both AI and HI plans, yielding paired measurements for each implant (Figure 2d).

#### 2.6.3 Bone Thickness Around Implants

**FIGURE 3:**
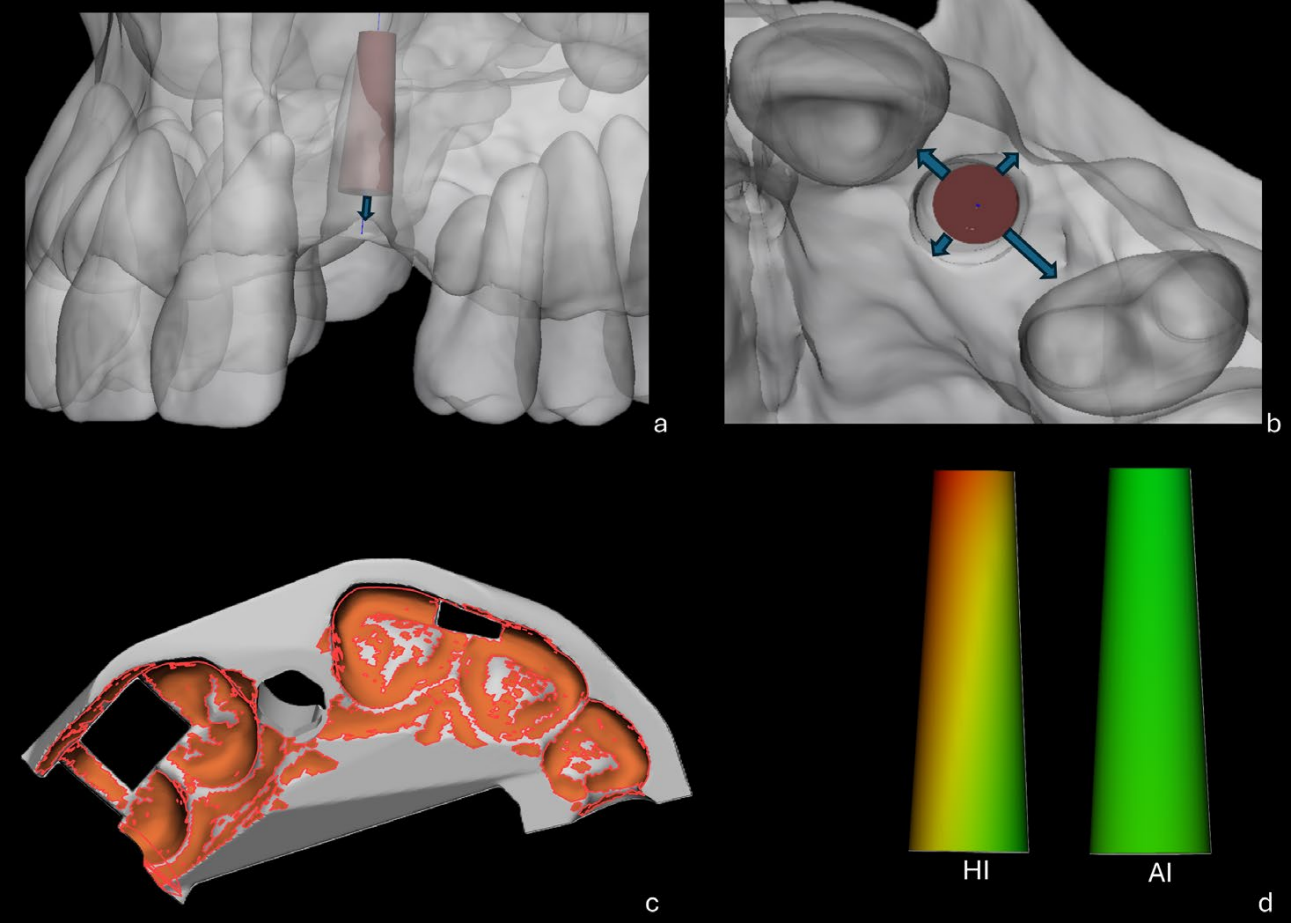
Performance assessment of AI‐driven implant planning: (a) Measurements of distance from the implant platform to the crestal bone; (b) Labial and palatal bone thickness and distance to adjacent teeth; (c) Surgical guide fit represented by the surface area of contact between the guide and IOS (orange areas); (d) Consistency assessment based on surface deviation between first and second plans for both HI and AI, Green color indicates perfect alignment; yellow, orange, and red colors indicate increasing deviation.

The AI‐segmented maxilla and teeth derived from CBCT data were fused with IOS‐derived crown data to create a unified model, which was then imported into the 3‐Matic software program. Bone thickness was measured in millimeters from the crestal, labial, palatal, mesial, and distal bone surfaces to the implant platform for each implant to evaluate differences in bone support between planning approaches (Figure 3a,b).

#### 2.6.4 Implant Dimension Comparison

The selection of implant dimensions (diameter and length, in millimeters) was compared between the AI and the HI for each missing tooth. For every implant site, the implant size proposed by the AI was recorded alongside the corresponding HI selection, and these paired measurements were used to quantify the degree of difference between AI‐driven and human‐derived choices in determining the most appropriate implant dimensions.

#### 2.6.5 Wax‐Up Evaluation

The planned implant STL models and their corresponding wax‐ups were subsequently imported into 3‐Matic software program for detailed analysis. Quantitative comparisons between HI‐ and AI‐based designs were performed by measuring (1) the angle between the automatically determined long axis of the planned implants and the long axis of the corresponding wax‐up, and (2) the distance between the wax‐up and the implant platform (Figure 2c).

#### 2.6.6 Surgical Guide Fit on the Virtual Model

The exported STL files of the surgical guides from both platforms were imported into 3‐Matic software program for analysis. The fit of each guide on the original IOS scans was evaluated using the software's collision detection tool, with a clearance threshold of 0.15 mm, corresponding to the designed offset of the guide from the teeth. The total contact surface area between the surgical guide and the IOS was quantified in millimeters squared (mm^2^) to evaluate the guide's overall adaptation (Figure 3c).

#### 2.6.7 Time Efficiency

The planning time for each method was recorded in seconds using a digital stopwatch to evaluate the total workflow efficiency. Timing started upon uploading the CBCT and IOS data and ended when the 3D implant positioning and final surgical guide design were completed. For both the AI‐assisted and HI approaches, the process was considered complete once the final plan and associated surgical template were saved for export, ensuring full capture of the prosthetically driven planning phase.

#### 2.6.8 Consistency Assessment

To assess the consistency of implant planning, 20% of the cases were randomly selected. For each approach (HI and AI), implant planning was repeated by the same operator who performed the initial planning procedure. To mitigate the risk of recall bias, a 2‐week washout period was maintained between the planning sessions. In the second session, CBCT scans and digital models were re‐coded and presented in a different random order. Clinicians were blinded to their previous plans, including implant dimensions and 3D positioning, so that each assessment was performed independently. The consistency between the two plans was evaluated by calculating the median surface deviation (MSD) and root mean square deviation (RMS). MSD reflects the average distance between corresponding points on two compared surfaces, whereas RMS quantifies the overall magnitude of misalignment. A value of zero millimeters for both parameters indicates complete positional agreement. The evaluation criteria for implant location assessment are illustrated in three dimensions in (Figure 3d).

### 2.7 Statistical Analysis

Statistical analyses were performed using R Studio software program (version 2026.01.0; Posit Software, PBC Boston, USA). Data normality was assessed using the Shapiro–Wilk test. Parametric data are presented as mean ± standard deviation (SD), whereas nonparametric data are reported as median and interquartile range (IQR). IQR values were presented using the 25th–75th percentiles. Linear deviations at the coronal and apical levels, as well as angular deviations, were summarized using descriptive statistics with corresponding 95% confidence intervals (CIs).

Reliability and absolute agreement between AI and HI planning were assessed using the intraclass correlation coefficient (ICC). ICC values were interpreted as poor (< 0.50), moderate (0.50–0.75), good (0.75–0.90), or excellent (> 0.90) (Koo and Li 2016). 95% CIs were calculated using the *F* distribution. Systematic bias and agreement limits were evaluated using Bland–Altman analysis, calculating mean differences and 95% limits of agreement (LoA; mean difference ± 1.96 × SD) for each coordinate (Yi et al. 2022). Hotelling's *T* ^2^ test was applied to compare multivariate mean vectors of the (X, Y, Z) coordinates at apical and coronal levels, assessing overall 3D differences between the implant planning methods (Hotelling 1931; Gerke 2020; Yi et al. 2022).

Paired comparisons between AI and HI were conducted based on data distribution. Nonparametric paired variables, including labial and palatal bone thicknesses and distances to adjacent teeth and crestal bone, implant size, time, surgical guide fit, RMS, and MSD were analyzed using the Wilcoxon signed‐rank test. Parametric paired variables, including crown to implant angle and distance from wax up to the implant platform, were analyzed using paired‐sample Student's *t*‐tests. A 5% significance level (α = 0.05) was applied to all analyses.

## 3 Results

### 3.1 Performance of Implant Position Selection

#### 3.1.1 Linear and Angular Deviation

Table 1 summarizes the descriptive comparison of HI and AI implant planning in terms of coronal, apical, and angular deviations. Using established clinical accuracy thresholds (coronal and apical deviations < 2.0 mm; angular deviation < 10°) (Van Assche et al. 2007; Schneider et al. 2009; Tahmaseb et al. 2014, 2018; Rungcharassaeng et al. 2015; Khaohoen et al. 2024; Song et al. 2025), mean coronal deviation was 0.96 ± 0.60 mm (median: 0.80 mm; 95% CI: 0.76–1.17 mm), with deviations ≥ 2 mm observed in 5.7% of cases, meeting clinical standards in 94.3% of implants. Apical deviation was higher, averaging 1.24 ± 0.66 mm (median: 1.20 mm; 95% CI: 1.00–1.50 mm), exceeding 2 mm in 14.3% of cases and meeting thresholds in 85.7% of implants. Angular deviation averaged 4.3° ± 2.9° (median: 4.2°; 95% CI: 3.4°–5.3°), with deviations ≥ 10° in 5.7% of cases, and met clinical standards in 94.3% of implants. One apical outlier reached 3.1 mm, representing the maximum observed deviation but remaining within ranges reported in the literature. Overall, 97.1% of implants achieved acceptable accuracy in at least two of the three evaluated dimensions. Deviations occurred predominantly at the apical level.

**TABLE 1:** Descriptive analysis of coronal, apical, and angular deviation between human intelligence and artificial intelligence planned dental implants.

	Mean ± SD	Median	95% CI for the mean	*n*	% implants ≥ 2 mm
Coronal deviation (mm)	0.96 ± 0.6	0.8	0.76–1.17	35	5.7%
Apical deviation (mm)	1.24 ± 0.66	1.2	1–1.5	35	14.3%

	Mean ± SD	Median	95% CI for the mean	*n*	% implants ≥ 10°
Angular deviation (°)	4.3 ± 2.9	4.2	3.4–5.3	35	5.7%

Abbreviations: %, percentage; °, degrees; CI, confidence interval; mm, millimeters; SD, standard deviation.

#### 3.1.2 Implant Planning Agreement Between AI and HI

**FIGURE 4:**
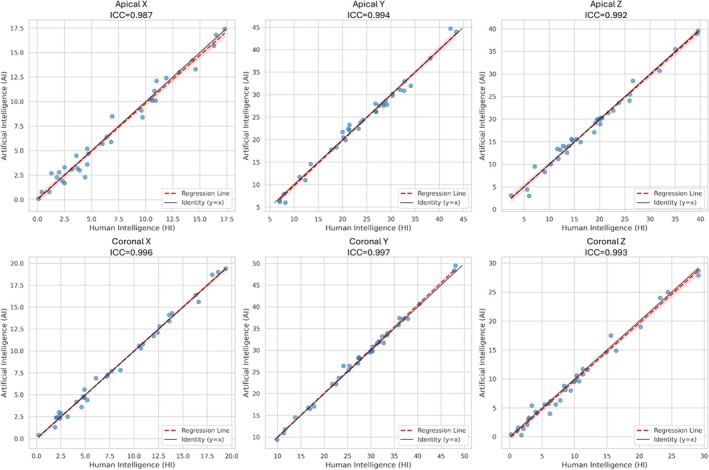
Correlation and Consistency Analysis of AI vs. HI Implant Planning. Scatter plots illustrate the relationship between AI and HI planning methods for apical and coronal coordinates (X, Y, and Z). Each panel displays the individual data points (*n* = 35), a linear regression line (red dashed), and the identity line (y = x, solid black) representing perfect agreement. The Intraclass Correlation Coefficient (ICC) values are provided for each coordinate, demonstrating excellent consistency across all spatial parameters (ICC ≥ 0.9).

ICC analysis demonstrated excellent agreement across all six spatial coordinates (Figure 4), with values above 0.9 and narrow 95% confidence intervals. All ICC estimates were statistically significant (*p* < 0.001), indicating high consistency between AI‐ and HI‐based implant planning. Effect size analysis showed negligible differences for five coordinates and a small effect for the coronal Y coordinate, suggesting that observed discrepancies were not clinically meaningful.

**FIGURE 5:**
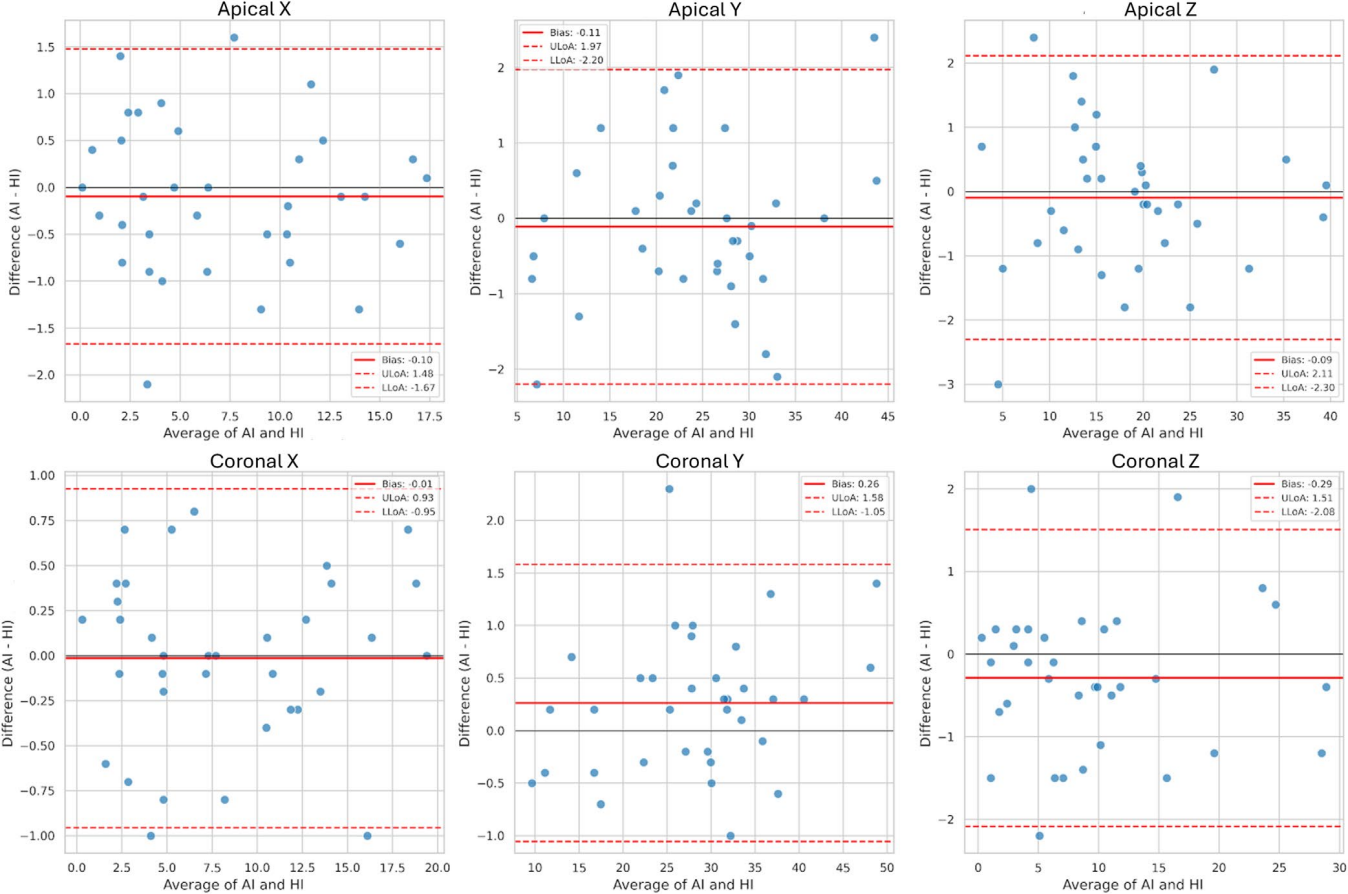
Bland–Altman Plots Evaluating Agreement and Bias. Bland–Altman plots representing the difference between AI and HI planning (AI—HI) plotted against their mean. The solid red horizontal line indicates the mean bias (average difference), while the dashed red lines represent the 95% Limits of Agreement (LoA; ±1.96 SD). The proximity of the mean bias to the zero‐reference line (solid black) suggests minimal systematic error, while the narrow LoA indicates high precision between the two planning approaches.

Bland–Altman analysis demonstrated minimal bias between the two methods for all coordinates (Figure 5), with absolute mean differences below 0.25 mm. The 95% limits of agreement ranged from approximately ±0.95 to ±1.82 mm and remained within clinically acceptable thresholds for implant positioning. Differences were symmetrically distributed around zero, with no evidence of proportional bias.

Multivariate testing using Hotelling's *T* ^2^ demonstrated no statistically significant multivariate difference in the 3D spatial coordinates (x, y, z) of the coronal and apical central points of implants planned using AI and HI. The analysis yielded a test statistic of $T^{2}=1.1765$ with 6 and 63 degrees of freedom for the numerator and denominator, respectively (*p* = 0.981). These findings indicate a high level of concordance between AI‐ and HI‐derived implant positions, confirming comparable geometric accuracy between both planning methods.

#### 3.1.3 Relation to the Surrounding Structures

Table 2 presents a comparative analysis of implant positioning within the alveolar bone for the HI and AI workflows. Evaluated parameters included labial and palatal bone thickness, distance to the crestal bone, and proximity to adjacent mesial and distal teeth. No statistically significant differences were detected between the two planning approaches for any parameter (*p* > 0.05), indicating comparable implant positioning relative to both the alveolar bone and neighboring dentition.

**TABLE 2:** Bone thickness and distance to neighboring teeth for HI‐ and AI‐planned dental implants.

	Distance to crest (mm)	Distance to buccal (mm)	Distance to palatal (mm)	Distance to mesial (mm)	Distance to distal (mm)
	Median (IQR)				
AI	1.7 (1.2–2)	2 (1.4–2.5)	1.3 (1–1.6)	2.7 (2.2–4)	2.8 (2–3.4)
HI	1.7 (1.4–2.2)	2 (1.7–2.5)	1.3 (1–1.7)	3 (2.1–3.6)	2.3 (2.1–3.2)
*p*	0.43	0.44	0.52	0.37	0.62

Abbreviations: AI, artificial intelligence; HI, human intelligence; IQR, interquartile range; mm, millimeters.

#### 3.1.4 Performance of Implant Size Selection

Table 3 shows that implant diameter and length selections were comparable between AI and HI, with no statistically significant differences in implant dimensions (*p* > 0.05). The median implant diameter was 3.8 mm in both methods (AI: IQR 3.3–3.8 mm; HI: IQR 3.1–3.8 mm), and the median implant length was 12 mm (AI: IQR 11.3–12.0 mm; HI: IQR 12.0–12.0 mm), indicating that the AI‐selected implant sizes closely matched those chosen by human clinicians.

**TABLE 3:** Assessment of planning time, surgical guide fit, and consistency based on median surface deviation (MSD), root mean square error (RMS) between human and AI planners.

	Consistency		Time (s)	Surgical guide fit (mm^2^)	Implant diameter (mm)	Implant length (mm)
	MSD (mm)	RMS (mm)				
	Median (IQR)					
AI	0 (0)	0 (0)	246 (200–365)	512 (437–566)	3.8 (3.3–3.8)	12 (11.3–12)
Human	0.7 (0.54–0.86)	0.9 (0.7–1.1)	420 (362–534)	526 (434–621)	3.8 (3.1–3.8)	12 (12–12)
*p*	< 0.05*	< 0.05*	0.0001*	0.21	0.27	0.93

*Note:* Asterisk (*) means statistically significant inference between the two groups.

Abbreviations: AI, artificial intelligence; HI, human intelligence; IQR, interquartile range; MSD, median surface deviation.

### 3.2 Wax‐Up Assessment

Table 4 summarizes the comparative evaluation of AI‐ and HI‐designed wax‐ups relative to the planned implant positions. No statistically significant differences were observed between the two approaches in either the crown–implant angle or the distance to the implant platform. The crown–implant angle showed comparable mean values for AI (11.7° ± 5.3°; 95% CI: 9.9°–13.5°) and HI (11.8° ± 5.7°; 95% CI: 9.9°–13.7°) assessments (*p* = 0.94). Likewise, the mean distance to the implant platform was identical for both methods (3.7 ± 1.2 mm for AI vs. 3.7 ± 1.4 mm for HI), with overlapping 95% confidence intervals (AI: 3.3–4.1 mm; HI: 3.2–4.1 mm) and no significant difference (*p* = 0.84).

**TABLE 4:** Assessment of wax‐up designs by HI and AI planners and their alignment with planned implants.

	AI	HI	*p*
Crown to implant angle, Mean ± SD	11.7 ± 5.3	11.8 ± 5.7	0.94
Crown to implant angle, 95% CI for mean	9.9–13.5	9.9–13.7	
Distance to implant platform, Mean ± SD	3.7 ± 1.2	3.7 ± 1.4	0.84
Distance to implant platform, 95% CI for mean	3.3–4.1	3.2–4.1	

Abbreviations: AI, artificial intelligence; CI, confidence interval; HI, human intelligence; SD, standard deviation.

### 3.3 Surgical Guide Fit

No statistically significant difference (V = 225, *p* = 0.21) was found in surgical guide fit on the IOS models between the AI and HI workflows (Table 3). The median surface contact area was 512 mm^2^ (IQR 437–566) for AI‐planned guides and 526 mm^2^ (IQR 434–621) for HI‐planned guides.

### 3.4 Time Assessment

AI‐based implant planning required significantly less time than human planning, with a median of 246 s (IQR 200–365) versus 420 s (IQR: 362–534) for HI (*V* = 18, *p* = 0.0001) (Table 3).

### 3.5 Consistency Assessment

Consistency analysis of repeated implant planning (Table 3) revealed significant differences between HI and AI workflows. HI plans demonstrated a median (IQR) MSD of 0.7 (0.54–0.86) mm and RMS of 0.9 (0.7–1.1) mm, whereas AI plans showed no deviation for either measure (*p* < 0.05), indicating superior reproducibility of AI‐based planning.

## 4 Discussion

The integration of AI into the digital implant dentistry workflow has shown considerable promise in enhancing presurgical implant planning, from CBCT‐based segmentation to subsequent IOS to CBCT registration (Preda et al. 2022; Morgan et al. 2022; Ayidh Alqahtani et al. 2023; Oliveira‐Santos et al. 2023; Wang et al. 2024; Elgarba, Ali, Fontenele, et al. 2025; Elgarba, Fontenele, Du, et al. 2025). Furthermore, AI can streamline implant location and dimension selection to support treatment decision‐making by reducing processing time and minimizing inter‐operator variability (Elgarba, Fontenele, Mangano, and Jacobs 2024; Elgarba et al. 2026; Dawood, Abumaylih, et al. 2026). Nonetheless, clinical outcomes remain site‐specific, as each region of the oral cavity is characterized by distinct anatomical features and treatment requirements. Traditionally, implant planning requires multidisciplinary collaboration among radiologists, surgeons, periodontists, and prosthodontists, which is advantageous but time‐consuming (Elgarba, Fontenele, Tarce, and Jacobs 2024).

This study presented a novel evaluation of AI‐assisted implant planning in the maxillary esthetic anterior zone. This region is particularly complex due to its stringent esthetic and functional requirements, which make the optimal positioning of implants particularly challenging. As emphasized by Flügge et al. (2022), this anatomical area poses substantial challenges to achieving consistent, high‐quality outcomes for dental implants. By reducing operator‐dependent variability and increasing planning efficiency, the integration of AI has the potential to promote greater predictability and standardization in both esthetic and functional results in routine clinical practice. In this study, AI was used as an initial planning support tool, generating preliminary implant positioning proposals that were subsequently reviewed and validated by experienced clinicians to ensure clinical accuracy and relevance.

In this study, the AI was trained to maintain a 2 mm labial and 1 mm palatal bone buffer, a critical threshold for ensuring both esthetic stability and soft tissue health. Our results demonstrate that the AI‐generated plans successfully met these parameters, yielding median bone thicknesses of 2.0 mm labially and 1.5 mm palatally. These values were statistically comparable to the HI plans. These findings align with established clinical guidelines. For instance, Rojas‐Vizcaya (2013) showed that maintaining 2 mm of labial bone requires placing the implant 2 mm palatal to the cervical contour of the adjacent teeth; meanwhile, Cicciù et al. (2023) demonstrated that a bone envelope exceeding 2 mm labially and 1 mm palatally effectively reduces the risk of peri‐implant bone loss.

The 3D accuracy of the AI‐assisted planning proposals demonstrated high performance relative to established clinical benchmarks. The mean coronal, apical, and angular deviations observed in this study were 0.96 ± 0.6 mm, 1.24 ± 0.7 mm, and 4.3° ± 2.9°, respectively. These results fall well within the “excellent” range defined by current literature, which typically sets thresholds at < 1.5 mm for linear and < 5° for angular deviations (Van Assche et al. 2007; Tahmaseb et al. 2018; Song et al. 2025; Dawood, Van Aelst, et al. 2026).

Our findings are consistent with previous systematic reviews and clinical validations. For instance, Tahmaseb et al. (2018) reported mean deviations of 1.12 mm (coronal), 1.39 mm (apical), and 3.89° (angular), while others have confirmed that linear deviations up to 2 mm and angular shifts near 5° remain clinically acceptable (Cretu et al. 2023; Takács et al. 2023).

While these results suggest that AI‐generated proposals achieve technical accuracy comparable to expert plans, this interpretation relies on the assumption that plan‐to‐plan concordance translates directly to equivalent clinical success. This surrogate endpoint, the relationship between planning accuracy and long‐term functional outcomes, remains to be validated through prospective clinical trials.

The excellent agreement across all spatial coordinates (ICC > 0.9) indicates that AI‐assisted planning can show close agreement with expert planning in determining implant position. The minimal deviations observed in the Bland–Altman analysis (< 0.25 mm) confirm that differences between AI and clinician planning fall well within clinically acceptable limits, suggesting a theoretically negligible influence on prosthetic fit or biological outcomes. The absence of proportional or systematic bias across apical and coronal levels using Hotelling's *T* ^2^ further demonstrates that AI does not introduce directional errors that could compromise esthetics or biomechanics (Yi et al. 2022). Taken together, these findings substantiate the clinical reliability of AI‐assisted workflows, supporting their use as effective decision‐making aids for precise and predictable implant placement in routine practice.

The present study found comparable mean wax‐up‐to‐implant angles for AI‐assisted (11.7° ± 5.3°) and HI (11.8° ± 5.7°) planning, indicating that AI can generate planning outputs that are closely aligned with those of experienced clinicians and support favorable implant‐to‐crown alignment. This finding is particularly relevant in the maxillary anterior region, where ideal implant trajectories are often constrained by tooth prominence and alveolar bone morphology, frequently necessitating minor angulation adjustments to maintain proper prosthetic orientation (Shi et al. 2021; Edmondson et al. 2022). The observed angulations in both groups remained well within clinically acceptable limits, generally considered to be up to 15° for anterior implants, beyond which biomechanical stress and biological complications, including peri‐implantitis, may become more likely (Dawood et al. 2021; De Angelis et al. 2025; Misch et al. 2025; Kaur et al. 2025).

Nevertheless, even small deviations may influence prosthetic design. Angulations below 15° generally favor screw‐retained restorations by allowing screw access through the cingulum or palatal surface, thereby preserving esthetics and retrievability (Edmondson et al. 2022). In cases of greater deviation or anatomical constraints, angled screw channel abutments may be required to redirect the access opening, while cement‐retained restorations may be considered to optimize the emergence profile and avoid visible buccal access holes (Sakamoto et al. 2018; Kraus et al. 2022). However, cemented options require careful management because of the risk of residual cement‐related complications (Dini et al. 2021). These findings suggest that AI‐assisted planning may support clinically reliable and prosthetically favorable implant placement in the esthetic anterior maxilla.

The vertical relationship between the prosthetic crown and the implant platform is a critical determinant of emergence profile development, soft tissue support, and long‐term esthetic stability (Spinato et al. 2022; Alrmali et al. 2023). Maintaining a distance of 3–4 mm from the gingival margin of the proposed crown to the implant platform is considered optimal for biological width formation and natural prosthetic appearance (Grunder et al. 2005; Springer et al. 2025).

In this study, the AI‐planned implants achieved a mean distance of 3.7 ± 1.2 mm, which closely matched the HI plans (3.7 ± 1.4 mm). Both workflows remained within the clinically preferred range. Positions below 3 mm significantly increase the risk of soft tissue recession, while distances exceeding 4 mm may necessitate excessive prosthetic buildup and adversely affect crestal bone stress (Esquivel et al. 2021; Ceddia et al. 2025; Dawood et al. 2026a).

Static guided surgery demonstrates effectiveness for precise implant placement. This study revealed comparable surgical guide fit on virtual models between HI and AI‐planned cases, with median surface contact areas of 512 (129) mm^2^ for AI and 526 (187) mm^2^ for HI. These findings suggest that AI‐assisted guide design may support efficient and accurate guide fabrication (Gupta et al. 2025). Recent reviews confirm that AI models in implant dentistry achieve high performance, with a mean accuracy of 88.7%, highlighting clinical applicability and workflow streamlining potential (Andrade‐Bortoletto et al. 2025).

Recent studies underscore AI's clinical value in improving time efficiency and consistency. AI‐driven tools significantly reduce planning time to 187–198 s compared to 406–435 s for human experts (Elgarba, Fontenele, Mangano, and Jacobs 2024; Elgarba, Fontenele, Du, et al. 2025) and from 30 to 10 min per case (Satapathy et al. 2024). This study demonstrated that AI‐assisted planning reduced the overall planning time by approximately 40% compared with HI planning, confirming a substantially faster and more efficient workflow. Notably, these time advantages are likely even more pronounced for novice operators, who typically require more time for implant planning due to the learning curve associated with implant positioning, prosthetic alignment, and anatomical interpretation. In contrast, AI provides rapid, standardized outputs independent of the operator's clinical experience (Rungcharassaeng et al. 2015; Elgarba, Fontenele, Du, et al. 2025; Elgarba, Ali, Fontenele, et al. 2025).

AI also demonstrates superior consistency, with AI plans showing zero deviation in repeated planning (MSD 0 mm, RMS 0 mm) compared to HI plans (MSD 0.7 ± 0.32 mm, RMS 0.9 ± 0.41 mm). These findings underscore AI's capacity to enhance planning efficiency and reproducibility while maintaining high clinical accuracy (Elgarba, Fontenele, Mangano, and Jacobs 2024; Ntovas et al. 2025).

This study demonstrated AI‐based virtual implant planning in the esthetic maxillary zone, achieving optimized implant positioning with improved efficiency and accuracy. Nonetheless, certain limitations should be noted. The analysis was restricted to maxillary anterior sites with adequate bone volume and relied on standardized datasets acquired using a single CBCT and IOS system. As image quality and dimensional accuracy can vary significantly across different scanner brands and acquisition protocols, these factors may limit the generalizability of the present findings (Fontenele et al. 2025; Gaêta‐Araujo et al. 2025).

Furthermore, this study reflects a comparison of the workflows, where overall clinical performance remains the focus (Fontenele et al. 2025; Papasratorn et al. 2025). However, it must be acknowledged that this study compares two distinct software platforms; therefore, platform‐specific effects (e.g., user interface, rendering algorithms, and integrated planning tools) cannot be fully separated from differences between AI‐assisted and HI planning (Strauss et al. 2024). Additionally, surgical guide validation was limited to an in vitro assessment, and future in vivo studies are needed to confirm clinical performance and patient‐centered outcomes.

The generalizability of these AI‐driven outcomes must be evaluated within the context of the noted anatomical variations of the maxillary anterior segment. The maxillary anterior region is anatomically heterogeneous, and central incisors, lateral incisors, and canines present distinct planning challenges (Gerhardt et al. 2022). Central incisors are often influenced by nasopalatine anatomy and midline spatial constraints; lateral incisors may exhibit greater crown and root morphology variability with reduced mesiodistal space; and canines are typically associated with longer roots, arch curvature, and labial undercuts. Accordingly, the present findings should be interpreted as applicable to anterior maxillary single‐tooth planning under the studied conditions, while tooth‐specific validation studies are still required to confirm generalizability across individual anterior sites.

Future multicenter studies with larger, tooth‐specific cohorts are needed to confirm reproducibility across diverse bone qualities and imaging systems, while extending AI models to incorporate soft‐tissue analysis, gingival biotype, and emergence profile prediction to fully meet anterior esthetic demands.

## 5 Conclusion

AI‐assisted implant planning in the esthetic maxillary anterior zone demonstrated promising performance, which is comparable to human expert planning, while offering advantages in time efficiency and planning consistency. The AI system achieved coronal, apical, and angular deviations within established clinical thresholds in the majority of cases, and the resulting surgical guide designs were comparable to those produced by human experts. These findings suggest that AI tools may serve as valuable decision‐support systems to enhance the efficiency and standardization of implant planning workflows.

## Author Contributions

**Rocharles Cavalcante Fontenele:** writing – review and editing, visualization, investigation. **Eslam Abdelwahab Dawood:** conceptualization, methodology, resources, data curation, visualization, investigation, writing – original draft. **Reinhilde Jacobs:** conceptualization, methodology, supervision, writing – review and editing. **Bahaaeldeen M. Elgarba:** conceptualization, investigation, methodology, data curation, resources, visualization, writing – review and editing.

## Funding

The researcher (Eslam Abdelwahab Dawood) is funded by a full scholarship (GM56‐2021) from the Ministry of Higher Education of the Arab Republic of Egypt.

## Ethics Statement

This study was approved by the Local Ethics Committee (reference number: S66447) and adhered to the principles outlined in the International Conference on Harmonization Guidelines for Good Clinical Practice, the Declaration of Helsinki, the Oviedo Convention, and relevant legal requirements (World Medical Association 2013). All patient data were anonymized to ensure confidentiality.

## Conflicts of Interest

The authors declare no conflicts of interest.

## Supporting information


**Data S1:** clr70144‐sup‐0001‐supinfo.docx.

## Data Availability

The data that support the findings of this study are available on request from the corresponding author. The data are not publicly available due to privacy or ethical restrictions.
